# Noninvasive Brain Stimulation Enhances Memory Acquisition and Is Associated with Synaptoneurosome Modification in the Rat Hippocampus

**DOI:** 10.1523/ENEURO.0311-19.2019

**Published:** 2019-12-03

**Authors:** Seung Ho Jung, Candice Hatcher-Solis, Raquel Moore, Naomi Bechmann, Sean Harshman, Jennifer Martin, Ryan Jankord

**Affiliations:** 1 Applied Neuroscience Branch, 711th Human Performance Wing, Air Force Research Laboratory, Wright-Patterson AFB, OH 45433; 2 Research Associateship Program, National Research Council, National Academies of Science, Washington, DC 200001; 3 ORISE, Oak Ridge, TN 37830; 4 Infoscitex, Dayton, OH 45431; 5 UES, Inc, Dayton, OH 45432; 6 Human Signatures Branch, 711th Human Performance Wing, Air Force Research Laboratory, Wright-Patterson AFB, OH 45433

**Keywords:** hippocampal proteomics, hippocampal synaptoneurosome, memory process, protein sequence similarity network, protein-protein interaction network, transcranial direct current stimulation

## Abstract

Transcranial direct-current stimulation (tDCS) is a non-invasive brain stimulation approach previously shown to enhance memory acquisition, but more studies are needed to elucidate the underlying mechanisms. Here, we examined the effects of anodal tDCS (0.25 mA for 30 min) on the memory performance of male Sprague Dawley rats in the passive avoidance test (PAT) and the associated modifications to the hippocampal proteomes. Results indicate anodal tDCS applied before the acquisition period significantly enhanced memory performance in the PAT. Following PAT, synaptoneurosomes were biochemically purified from the hippocampi of tDCS-treated or sham-treated rats and individual protein abundances were determined by bottom-up liquid chromatography mass spectrometry analysis. Proteomic analysis identified 184 differentially expressed hippocampal proteins when comparing the sham to the tDCS before memory acquisition treatment group. Ingenuity pathway analysis (IPA) showed anodal tDCS before memory acquisition significantly enhanced pathways associated with memory, cognition, learning, transmission, neuritogenesis, and long-term potentiation (LTP). IPA identified significant upstream regulators including *bdnf*, *shank3*, and *gsk3b*. Protein-protein interaction (PPI) and protein sequence similarity (PSS) networks show that glutamate receptor pathways, ion channel activity, memory, learning, cognition, and long-term memory were significantly associated with anodal tDCS. Centrality measures from both networks identified key proteins including *dlg*, *shank*, *grin*, and *gria* that were significantly modified by tDCS applied before the acquisition period. Together, our results provide descriptive molecular evidence that anodal tDCS enhances memory performance in the PAT by modifying hippocampal synaptic plasticity related proteins.

## Significance Statement

We investigated whether anodal transcranial direct-current stimulation (tDCS) affects memory performance and the underlying protein modifications in hippocampal synaptoneurosomes. We found that anodal tDCS administered before memory acquisition significantly enhanced memory performance by enhancing the expression of hippocampal proteins associated with glutamate signaling and ion channel activity. Our results identify molecular targets for tDCS-induced memory enhancement and the associated signaling pathways. Our behavioral and proteomics study further elucidates the mechanism for tDCS effects on acquisition memory and may lead to the development of therapeutics to enhance memory and learning process to treat for neurologic diseases and psychological disorders.

## Introduction

Transcranial direct-current stimulation (tDCS) is widely used clinically due to its non-invasive application and few reported side effects (Stagg and Nitsche, 2011; Holmes et al., 2016). Clinical studies have revealed the beneficial effects of tDCS as a therapeutic tool for neurologic diseases and psychological disorders including Alzheimer’s disease, schizophrenia, depression, and anxiety (Marshall et al., 2004; Boggio et al., 2006; Göder et al., 2013; Ladenbauer et al., 2017; Hill et al., 2019; Medvedeva et al., 2019). Although the biological and molecular mechanisms remain unclear, tDCS has been shown to enhance memory. tDCS improves working memory (Hoy et al., 2013; Martin et al., 2014; Bogdanov and Schwabe, 2016), long-term memory (Javadi and Cheng, 2013), semantic memory (Cattaneo et al., 2011; Holland et al., 2011), and memory acquisition (Jacobson et al., 2012; Javadi and Walsh, 2012). Moreover, a study using elderly subjects showed that anodal tDCS significantly enhanced memory recall one week after learning compared to sham stimulation, suggesting long lasting effects of tDCS (Flöel et al., 2012). Although studies have shown that tDCS enhances memory, especially memory acquisition, there is limited biological data available for the underlying effects of tDCS on the hippocampal proteome, which may explain how tDCS induces memory enhancement.

Several studies in humans and animals have examined the effects of tDCS on neuronal activity and synaptic plasticity to understand the regulatory mechanisms. Electrophysiological studies have revealed that tDCS increases cortical excitability in humans (Alonzo et al., 2012; Romero Lauro et al., 2014; Bailey et al., 2016). Animal studies have shown that tDCS enhances synaptic plasticity in the hippocampus of rodents (Rohan et al., 2015; Podda et al., 2016). However, the molecular mechanisms of tDCS to enhance memory and learning are less known. tDCS has been shown to affect the expression of immediate early genes *c-fos* and *zif268* in the hippocampus (Ranieri et al., 2012). Studies have further determined that tDCS affects the mRNA level of hippocampal brain-derived neurotrophic factor (BDNF), a growth factor important for long-term memory (Podda et al., 2016; Kim et al., 2017). Additional studies indicate tDCS may regulate neurotransmitter signaling of glutamatergic, GABAergic and cholinergic pathways (Rossini et al., 2015; Giordano et al., 2017) that favor enhancement of memory and cognition. tDCS has also been shown to modify the expression of genes related to serotonergic, adrenergic, dopaminergic, GABAergic, and glutamatergic signaling in the rat cortical transcriptome (Holmes et al., 2016). A more recent study showed that tDCS modifies AMPA receptor phosphorylation and translocation in the rat hippocampus (Stafford et al., 2018), suggesting a possible effect of tDCS on protein modifications in rat hippocampal synaptoneurosomes. Another study reports that anodal tDCS enhances performance in the hippocampal-dependent passive avoidance memory task and that the tDCS-induced enhancement was abrogated with pretreatment of ANA-12, an inhibitor of the BDNF receptor tropomyosin receptor kinase B (Yu et al., 2019). More studies are needed to associate tDCS-induced effects on hippocampal protein regulation with behavioral performance to determine mechanism.

In this study, we examined whether anodal tDCS affected memory acquisition and/or recall along with the molecular modifications of stimulation on synaptic proteomics in the rat hippocampus. We show that anodal tDCS (250 μA for 30 min) applied before the memory acquisition period of a learning and memory test enhances cognitive performance. We report that the enhancement of memory acquisition by anodal tDCS is significantly related to molecular alterations in the hippocampal proteome. The tDCS-induced modifications in hippocampal synaptoneurosomes are significantly associated with receptor signaling and voltage-gated ion channel activity in pathways associated with learning, memory, and cognitive enhancement.

## Materials and Methods

### Animals

Adult Sprague Dawley rats (male, seven to eight weeks old weighing ∼400–500 g, *n* = 14/group) were purchased from Charles River Laboratories. Rats were housed in the animal facility of the Wright-Patterson Air Force Base (WPAFB) with ad libitum access to food and water and maintained on a 12/12 h light/dark cycle. Rats received a 10-d acclimation period before surgical electrode placement. All rats were maintained according to National Institutes of Health and WPAFB Institutional Animal Care and Use Committee guidelines. The study protocol was reviewed and approved in compliance with the Animal Welfare Act and with all applicable federal regulations governing the protection of animals in research.

### Surgical implantation of cranial electrode

Animals were anesthetized with isoflurane (Med-Vet International) using 5% induction, followed by 2–3% isoflurane to maintain anesthetic depth. A 5-mm diameter, circular, head electrode casing (Tangible Solutions) was attached to the skull from 0 to –5 mm bregma. Luting dental cement (GC Fuji I, GC America Inc.) was applied to the base of the head electrode casing and to the skull, followed by an acrylic dental cement (Sigma-Aldrich) to secure the electrode casing. Animals were given a minimum of 7 d as a recovery period before tDCS treatment. Rats were randomly selected for sham, anodal tDCS before acquisition, or anodal tDCS before memory recall.

### tDCS application

**Figure 1.**
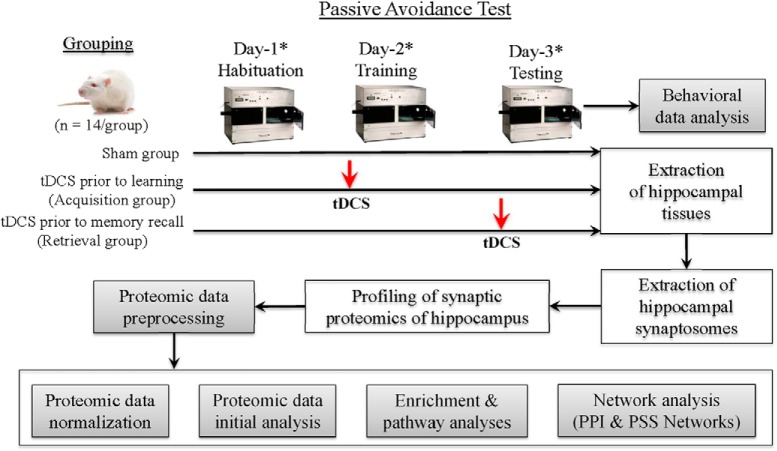
Overall research design. *Before rodents were exposed to the passive avoidance memory task, they were freely exposed to open field for 5 min (the acquisition day) and 3 min (the training and testing days) for exploration with familiar and novel objects similar to the novel object recognition task. Proteomic abundance data were first analyzed for the replicability within each group (Extended Data Fig. 1-1), and the abundance of 16 internal control proteins was compared between the groups (Extended Data Fig. 1-2). Proteomic data analyzed for this manuscript were provided as an Excel file (Extended Data Fig. 1-3).

On the same day, before stimulation, animals were acclimated to the testing room for 10 min. A conducting medium (SignaGel, Parker Laboratories) was placed into the head casing before connecting the head electrode. The reference electrode (12-mm diameter, Tangible Solutions) was placed on the rat’s shaved chest with SignaGel as the conducting medium. Once the electrodes were in place, the animal was wrapped with a flexible cohesive bandage (PetFlex, Med-Vet) and placed into their home cage. Anodal tDCS was then applied at 0.25 mA using a constant-current stimulator (Magstim DCstimulator; Neuroconn) for 30 min. The sham group was prepared the same way as the stimulation groups but did not receive any current (Fig. 1).

10.1523/ENEURO.0311-19.2019.f1-1 Extended Data Figure 1-1 Replicability of proteomic abundance data within each group. The abundance values within each group were analyzed and the lowest r2 for the sham, acquisition and retrieval groups were 0.89, 0.91, and 0.72, respectively. Download Figure 1-1, TIF file.

**Figure 2.**
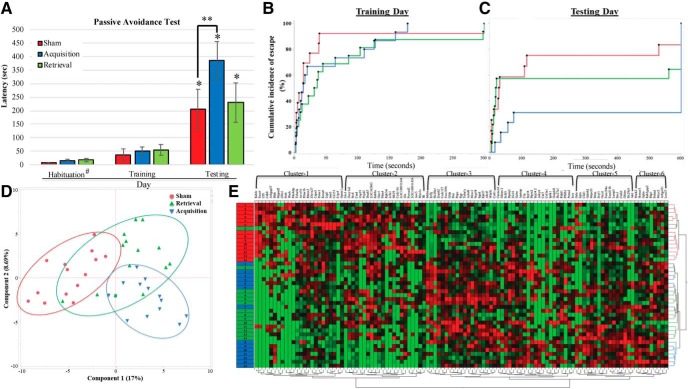
Behavioral data analysis. ***A***, Analysis of passive avoidance data. Two-way RM ANOVA detected a significant difference in the latency of all groups between the training and testing days. During the testing day, a statistical significance was detected between sham and acquisition groups. #Habituation data were collected from only eight animals per group; * and ** indicate statistical significance (*p* < 0.05 and *p* < 0.001, respectively). ***B***, Cox proportional hazard regression analysis for PAT data on training day. No statistical group separation was detected. ***C***, Cox proportional hazard regression analysis for PAT data on testing day. A significant group difference was detected (log-rank *Χ* ^2^ = 7.0919, df = 2, *p* = 0.0288). ***D***, PCA with proteomics data show the distribution of each samples across the groups. ***E***, Hierarchical clustering analysis with proteomics data show the distribution of each sample across the groups. Red, green, and blue boxes represent sham, retrieval, and acquisition samples. Protein IDs in the first hierarchical clustering group, from left to right, are KDELC2, EXOC6, UFL1, CDH8, ARHGAP27, BLNK, MPPED2, GFM1, FNTB, WASH, PDE4DIP, TUBA4A, ACVR1B, PRKAR1B, RBMX2, ZFYVE27, CAPN5, PSME1, KCNA6, LLGL1, JAM3, CD151, S1PR5, and GPSM1. The hierarchical clustering group 2 includes, from left to right, LHX1, GUCY1A2, STX8, CSAD, USP19, FTSJD2, DNAH7, LOC362863, RAB9A, GSTZ1, PDIA4, RPH3AL, LRFN1, STK39, CCDC116, LOC100911646, GLS, PYROXD2, LOC100911456, AOC3, MYO1C, and ILK. For the cluster group 3, protein IDs are INHBA, EIF3J, SLC20A1, ZRANB2, CCDC127, FAM195B, GLDN, FHIT, PNPO, PGP, TUBA1C, ACBD6, MCCC1, NOP58, ITGAD, RPL27, ABCF1, WDR81, and COTL1. The cluster group 4 contains proteins named as, from left to right, CACNA1D, GHITM2, SCFD1, PTP4A2, ZW10, GCLM, APMAP, CRP, SRP54, PTPRN2, SRP542, ADCY6, CAR3, COL3A1, IP6K1, GOLPH31, SLC27A1, RILPL1, PFKFB1, PSMA2, and ICOS. Proteins from the cluster group 5 are GALE, EQTN, CLUL1, GABRB2, MRPS25, MRAS, SCYL1, OXCT1, FAM194A, FAM213B, LAMP5, SNX1, CNIH2, GHITM, SLC29A3, and AK6. Proteins in the cluster group 6 are MARK2, HOMER3, GPHN, RAB3GAP2, RBP1, PALMD, MCCC2, ARHGEF11, and LRRC57. For a clear figure, see Extended Data Figure 2-1.

10.1523/ENEURO.0311-19.2019.f1-2 Extended Data Figure 1-2 Abundance comparison of 16 internal control proteins between groups. None of all 16 internal control proteins resulted in no significant difference between acquisition and sham groups. The comparison between retrieval and sham groups, one internal control protein (*Aldoa*) showed a significant group difference. The abundance data of three internal control proteins (*Aldoa*, *Gdir1*, and *Pgk1*) resulted in a significant group difference between acquisition and retrieval groups. Download Figure 1-2, TIF file.

**Figure 3.**
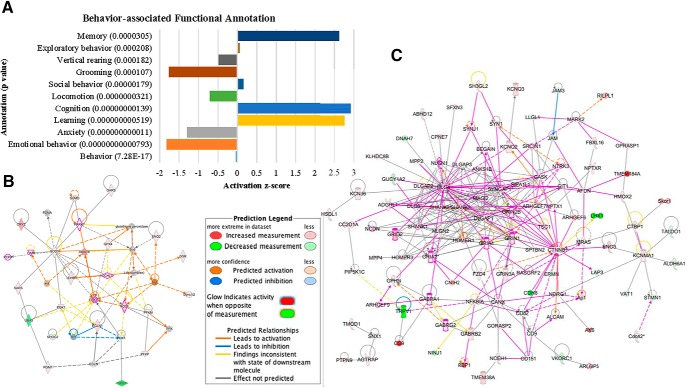
IPA results. ***A***, Behavior-associated functional annotation detected from the comparison between the acquisition and sham groups. ***B***, Network-1 detected from the comparison between the retrieval and sham groups. ***C***, Six networks that were associated with the nervous system were merged in to a network. Nodes that did not pass cutoff in dataset, not in overlaid dataset, and not connected to any other nodes were excluded from the network. Pink-outlined molecules are associated with cognition (*p* = 1.81E-3). Molecule activity predictor (MAP) was also overlaid to predict the upstream and downstream effects of activation or inhibition on other molecules. For all IPA data, see Extended Data Figures 3-1, 3-2, 3-3, 3-4.

10.1523/ENEURO.0311-19.2019.f1-3 Extended Data Figure 1-3 Proteomics data. Proteomics data analyzed for this manuscript were provided as an Excel file. Download Figure 1-3, XLSX file.

### Behavioral tests

To assess memory acquisition and recall following tDCS, a modified passive avoidance test (PAT) was run as a learning and memory test (Fig. 1). Before behavioral testing, animals were allowed to acclimate to the testing room for at least 10 min. On day 1 (habituation), animals had their midsection wrapped in Petflex tape and were returned to their home cage for 20 min. They were then unwrapped and placed into an open arena and allowed to explore freely for 5 min. Immediately following exploration, animals were then taken to the PAT chamber and allowed to freely explore both rooms for 5 min.

Approximately 24 h later (training, day 2), animals were wrapped for stimulation and placed in their homecage. Only the group designated to receive stimulation before memory acquisition received anodal tDCS, and the other two groups received sham stimulation. After the 30-min stimulation period, animals were unwrapped and placed in the open arena on the opposite wall facing away from the two identical objects present. They were allowed to freely explore the arena for a total duration of 3 min. They were then taken to the PAT chamber (Gemini, San Diego Instruments, Inc.) and placed into a lit room facing away from the gate that led to a dark chamber. After a 30-s acclimation period, the gate rose, and the animal was given the opportunity to cross over to the dark room. Once the animal crossed over, the gate was immediately closed, and after 3 s the rat received 0.75-mA shock to the feet for 1 s. After the shock was administered, the rat remained in the dark room for 30 s before being removed and returned to its homecage.

Approximately 24 h later (testing, day 3), the animals were wrapped for stimulation and placed in their homecage. Only rats designated to receive anodal tDCS before memory recall were stimulated, and all of the other rats received sham stimulation. After the stimulation period, animals were unwrapped and placed into the same arena as the previous 2 d and allowed to explore the arena for 3 min. During this phase, one of the objects was randomly replaced with a different object that the rodent had not been previously exposed to. The different object was placed and rotated between animals. After the 3-min exploration, the rodent was then placed into the PAT lit room chamber facing away from the gate and allowed to acclimate for 30 s. The gate separating the light room from the dark room was then lifted and the animal was again given the opportunity to cross into the dark room for a period of 10 min (600 s). After the animal either crossed over to the dark room or the 10 min maximum time was reached, the animal was removed from the chamber and immediately euthanized by decapitation for brain tissue collection.

### Tissue handling

Tissue preparation followed procedures described previously (Stafford et al., 2018). Briefly, hippocampal tissue from each rat was collected, frozen and stored at –80°C. The hippocampal tissues were prepared following the Syn-PER protein extraction method described by the manufacturer (Thermo Fisher Scientific), which yielded two protein fractions: cytosolic and synaptic fraction. The concentration of the synaptic proteins (synaptoneurosomes) was assessed for each sample in duplicates, using the bicinchoninic acid assay (Thermo Fisher Scientific) according to manufacturer’s instructions.

### Proteomic sample preparation

Synaptoneurosome isolations (15 μl) were suspended in 15 μl of 0.2% Rapigest surfactant (Waters) in 50 mM ammonium bicarbonate supplemented with MS-SAFE protease/phosphatase inhibitor cocktail (Sigma-Aldrich). Protein samples were reduced with dithiothreitol (5 mM final concentration, Sigma-Aldrich) at 95°C for 5 min. Samples were cooled and cysteines were alkylated with iodoacetamide (15 mM final concentration, Sigma-Aldrich) at ambient temperature for 30 min in the dark, followed by the protein digestion with 800 ng of sequencing grade modified trysin/Lys-C (Promega Corporation) at 37°C overnight with gentle shaking. Neat formic acid (Pierce, Thermo Fisher Scientific) was added to each sample (50% v/v) and samples were returned to 37°C for 30 min. Precipitated Rapigest was removed by removing the supernatant following centrifugation at 30,000 × *g* for 15 min. Centrifugation was repeated until no precipitate remained. Samples were dried in a speed vac and resuspended in 2% acetonitrile: 0.03% trifluoroacetic acid (TFA; aq, LC loading buffer). The 280-nm absorbance was used to estimate peptide concentration (Nanodrop) and samples were diluted to 0.5 μg/μl in LC loading buffer.

### Bottom-up liquid chromatography mass spectrometry (LC-MS/MS)

All separations were performed on a Dionex Ultimate 3000 RSLCnano liquid chromatography system (Thermo Fisher Scientific). Briefly, digested peptides (1 μg) were preconcentrated on a 5 μ, 100 Å, 300 μm × 5 mm C18 PepMap 100 trap column (Thermo Fisher Scientific) using LC loading buffer under isocratic conditions at 5 μl min^−1^ for 7.5 min. Peptides were reversed-phase separated on an Easy-Spray PepMap 3 μm, 100 Å, 75 μm × 15 cm column at 300 nl min^−1^. Mobile phases were 0.1% formic acid (aq, A) and 0.1% formic acid in acetonitrile (B, Optima MS Grade, Thermo Fisher Scientific). Analytical separations were conducted over 180 min at 3% B for 10 min, 30% B for 152 min, 40% B at 157 min followed by a 10 min wash at 90% B, and a 10-min equilibration at 3% B. Eluted peptides were introduced into an Orbitrap Fusion Lumos mass spectrometer equipped with Easy-Spray source operated at 2.2 kV (Thermo Fisher Scientific). MS^1^ scans were acquired at 120,000 resolution across 375–2000 m/z using the Easy-IC reagent (fluoranthene) for internal mass calibration. Precursors were selected based on a MS^(^*^n^* ^–1)^ scans and isolated for data-dependent MS*^n^* scans in the quadrapole operated with 1.2m/z isolation window. Fragments were generated by collision-induced dissociation (CID) with a 10-ms activation time and a 35% normalized collision energy for +2 to +7 precursor charges states in the ion trap using all available parallelizable time over 2-s cycles. Dynamic exclusion for MS*^n^* scans was set at a ±10 ppm mass tolerance with exclusion occurring after one time for 15 s.

### Proteomic data processing

Proteomic data were searched using the Proteome Discoverer software suite (v. 2.2) equipped with the Sequest HT search engine (Thermo Fisher Scientific). Briefly, tandem data were searched against the Uniprot reviewed Rattus norvegicus (as of 25Oct17) and the common Repository of Adventitious Proteins (cRAP) databases. Search parameters were as follows: MS tolerance 10 ppm, an MS*^n^* tolerance of 0.5 Da, and three allowed missed tryptic cleavages. Amino acid modifications searched were carbamidomethyl of cysteine (static), oxidation of methionine (dynamic), and acetylation of the N terminus (dynamic). Peptide spectral matches (PSMs) were evaluated using the target decoy PSM validator with a maximum Cn of 0.05 and a decoy database search with a target false discovery rate (FDR, *q* value) of 0.05 (relaxed) and 0.01 (strict). FDR was estimated with 0.05 (relaxed) and 0.01 (strict) thresholds. Precursor ion label free quantitation was performed on unique and razor peptides with retention time alignment of <10 min, mass tolerance 10 ppm, abundance based on intensity, and data normalized to total peptide amount. Normalized abundances were exported from Proteome Discoverer for further downstream analysis described below.

### Data analysis and statistical methods

Behavioral data from the PAT consisted of latency variables that were analyzed by two-way repeated measures ANOVA with the Holm–Sidak *post hoc* method and Cox proportional hazard regression test. A homoscedastic two-tailed Student’s *t* test was conducted to identify statistically differentially expressed proteins (DEPs) of normalized abundance variables between treatment groups. Principal component analysis (PCA) on the correlation matrix with the default estimation method was employed to statistically explain the structure of normalized abundance data across groups. Hierarchical clustering analysis with the distance method of Ward to calculate distances between all pairs of clusters was performed. For these data analyses, the statistical software SigmaPlot (version 12.3, Systat Software, Inc.), JMP Pro (version 13.2, SAS Institute Inc.), and Microsoft Excel 2013 were used.

The enrichment and pathway analysis on DEPs of the normalized abundance data sets were performed with the Panther (Protein ANalysis THrough Evolutionary Relationships) classification system (Mi et al., 2013) and the Database for Annotation, Visualization and Integrated Discovery (DAVID) Bioinformatics resources (Huang da et al., 2009). The DAVID Bioinformatics resources was also used to perform a functional annotation clustering analysis for DEPs of the normalized abundance data sets. The DEPs were also analyzed by the Ingenuity Pathway Analysis (IPA; QIAGEN) to identify the diseases and biological functions that are associated with behaviors. The Ingenuity Upstream Regulator analysis in IPA was performed to identify the upstream transcriptional regulators in addition to conducting the IPA’s network analysis.

Protein-protein interaction (PPI) network analysis for the organism Rattus norvegicus (network edge setting: molecular action) was conducted for DEPs by using the STRING database (Szklarczyk et al., 2017). The Markov cluster (MCL) algorithm with an inflation parameter of 3 was used if needed. Protein sequence similarity (PSS) network analysis for the organisms Rattus norvegicus was also performed. The BLASTP suite (BLASTP 2.8.0+) was used to search proteins and determine their sequence similarity. Cytoscape (version 3.7) was used to build PSS network visualization and conduct network analysis (Shannon et al., 2003). The Cytoscape plugin app ClusterONE was used to cluster with overlapping neighborhood expansion to detect potentially overlapping protein complexes from the interaction data (Nepusz et al., 2012). Centrality measures were calculated to identify critical proteins that play a central role in mediating interactions among given proteins in PPI and PSS networks (Jeong et al., 2001; Ashtiani et al., 2018) by using CytoNCA (Wang et al., 2012). The GeneMANIA was also used to identify the function of molecules in networks (Montojo et al., 2010; Warde-Farley et al., 2010) as needed.

### Data availability

Proteomics data are provided as Extended Data Figure 1-3.

## Results

### PAT

The PAT was used to examine the effect of anodal tDCS administered before memory acquisition or memory recall on cognitive performance. Two-way repeated measures ANOVA (session × group) was performed for PAT latency variable. A significant trial difference (*F* = 25.05, *p* < 0.001) was detected (Fig. 2*A*). All groups resulted in statistical significance between training and testing days, confirming that the learning experience of PAT was significantly completed by all animals across treatment groups. Additionally, on the testing day, the *post hoc* test shows that the acquisition group significantly performed better on PAT when compared to the sham group (*p* = 0.046), but no significant difference between sham and retrieval groups was detected. However, because the two-way repeated measures ANOVA did not detect a statistical significance from the treatment group variation (*F* = 1.695, *p* = 0.196), Cox proportional hazard regression test was also conducted for PAT data on training and testing days (Fig. 2*B*,*C*). The result first confirmed no group difference during the training day. However, on the testing day, there is a significant difference between the acquisition and sham groups indicating that brain stimulation before training enhances memory performance (χ^2^ *p* = 0.0445, risk ratio = 2.306331).

10.1523/ENEURO.0311-19.2019.f2-1 Extended Data Figure 2-1 Hierarchical clustering analysis with proteomics data show the distribution of each sample across the groups. Download Figure 2-1, TIF file.

### Overall results of hippocampal synaptic proteomics

Proteomics analysis was conducted to determine the underlying effects of brain stimulation on protein abundance in the rat hippocampus, an area of the brain associated with learning and memory. The proteomics data were first validated by comparing the protein abundances within each group (replicability; Extended Data Fig. 1-1) and analyzing the abundance levels of 16 proteins that are usually used as internal control (Extended Data Fig. 1-2). All 16 proteins show no group difference between sham and acquisition groups, and 15 proteins resulted in no difference between sham and retrieval groups. The abundance levels of 3687 proteins were detected, but 2909 proteins were used for further analyses because 778 proteins contain a value of zero for the most samples (≥35 samples out of 42 samples; Extended Data Fig. 1-3). When compared to the sham group, the acquisition and retrieval groups resulted in 184 and 82 significant proteins, respectively (*p* < 0.0500, two-tailed test). Interestingly, 431 proteins were significantly identified when the two tDCS groups were compared. PCA showed a clear group separation between sham and acquisition groups, but the retrieval group overlapped with the other groups (Fig. 2*D*). The hierarchical clustering analysis resulted in the similar pattern to the PCA result (Fig. 2*E*).

### Gene ontology (GO) enrichment analysis and pathway analysis (PantherDB and DAVID Bioinformatics)

GO enrichment analysis and pathway analysis were performed to investigate the effects of tDCS on synaptoneurosomes and pathways in the rat hippocampus. Using lists of significantly, DEPs between two groups, GO enrichment analysis was performed by using PantherDB and DAVID Bioinformatics. The analysis from the comparison between sham and retrieval groups resulted in only three cellular component (CC) and two pathways. The two pathways are TGF-β signaling pathway (P00052, FDR *q* = 0.026) and gonadotropin-releasing hormone receptor pathway (P06664, FDR *q* = 0.00223). The analysis of Panther GO slim with significant genes between sham and acquisition groups (Table 1) resulted in 12 CC, 20 biological processes (BPs), 16 molecular functions (MFs), and seven pathways. The GO slim results showed that the significant genes are significantly associated with neuronal synaptic membrane and protein complex (from CC results); neuron-neuron synaptic transmission and transport (from BP results); and significantly connected to the signaling pathways of glutamate receptor and voltage-gated ion channel activities (from MF results). The results of the Panther pathways analysis include glutamate receptor pathways (P00037, P00041, and P00039) and beta-adrenergic receptor pathways (P04378 and P04377). Moreover, the Reactome pathway analysis shows that the significant genes identified from the comparison between sham and acquisition groups are significantly involved in 20 different pathways (Extended Data Table 1-1). Among them, the most abundant pathways are receptor signaling, including NMDA receptor, glutamate receptor, and ion channels. From the significant genes between sham and retrieval groups, however, no significant pathways and GO slim terms for BP and MF were detected. Only three GO slim CCs were detected and they are: cytoplasm (GO:0005737, fold change (FC): 2.39), intracellular (GO:0005622, FC: 1.71), and cell part (GO:0044464, FC: 1.68) with *q* values from FDR analysis < 0.05. The significant gene list identified from the comparison between the acquisition and recall groups resulted in 20 CC, 42 BP, and 26 MF with a statistical significance from the FDR analysis (Table 2; Extended Data Table 2-1). The CC terms identified by the GO slim analysis includes postsynaptic membrane, synapse, SNARE complex, neuron projection and protein complex (*q* < 0.05). Growth, neuron-neuron synaptic transmission, and different transports and metabolisms were detected for the BP terms (*q* < 0.05); while glutamate receptor activity, SNAP receptor activity and multiple kinase activities were identified for the MF terms (*q* < 0.05).

**Table 1.** Results of PantherDB analysis for the comparison between sham and acquisition groups

PANTHER GO-Slim pathways		Over/Under	Fold enrichment	Raw *p* value	FDR (*q* value)
Cellular Component	Postsynaptic membrane (GO:0045211)	+	30.25	9.68E-11	3.10E-09
	Neuromuscular junction (GO:0031594)	+	25.21	3.67E-04	2.14E-03
	Synapse (GO:0045202)	+	12.67	2.09E-11	1.34E-09
	Dendrite (GO:0030425)	+	9.71	7.15E-07	9.15E-06
	Neuron projection (GO:0043005)	+	7.03	8.27E-09	1.77E-07
	Cell junction (GO:0030054)	+	6.92	3.10E-04	1.98E-03
	Cell projection (GO:0042995)	+	4.9	3.05E-07	4.88E-06
	Integral to membrane (GO:0016021)	+	2.69	3.03E-05	2.42E-04
	Plasma membrane (GO:0005886)	+	2.43	4.05E-06	3.70E-05
	Cytoskeleton (GO:0005856)	+	2.31	9.41E-03	4.63E-02
	Membrane (GO:0016020)	+	2.14	3.70E-06	3.94E-05
	Protein complex (GO:0043234)	+	2.1	0.0001	7.14E-04
Biological Process	Growth (GO:0040007)	+	44.12	9.25E-05	2.51E-03
	Asymmetric protein localization (GO:0008105)	+	35.29	1.58E-04	3.86E-03
	Muscle organ development (GO:0007517)	+	26.14	3.03E-07	1.85E-05
	Neuron-neuron synaptic transmission (GO:0007270)	+	13.07	1.27E-08	1.55E-06
	Pyrimidine nucleobase metabolic process (GO:0006206)	+	11.39	2.91E-03	3.55E-02
	Synaptic transmission (GO:0007268)	+	6.38	4.01E-11	9.80E-09
	Ion transport (GO:0006811)	+	4.48	2.13E-06	8.66E-05
	Cell-cell signaling (GO:0007267)	+	4.43	2.12E-08	1.72E-06
	Cytoskeleton organization (GO:0007010)	+	3.22	1.35E-03	2.20E-02
	Protein localization (GO:0008104)	+	2.88	1.20E-03	2.08E-02
	Transport (GO:0006810)	+	2.35	4.58E-06	1.60E-04
	Localization (GO:0051179)	+	2.34	9.21E-07	4.50E-05
	Neurological system process (GO:0050877)	+	2.11	3.25E-04	7.21E-03
	System process (GO:0003008)	+	2.05	4.42E-04	8.30E-03
	Phosphate-containing compound metabolic process (GO:0006796)	+	1.94	1.45E-03	2.09E-02
	Cellular component organization (GO:0016043)	+	1.76	2.71E-03	3.48E-02
	Cell communication (GO:0007154)	+	1.55	3.25E-03	3.77E-02
	Sensory perception of smell (GO:0007608)	-	<0.01	2.15E-03	2.92E-02
	Sensory perception of chemical stimulus (GO:0007606)	-	<0.01	1.44E-03	2.19E-02
	Sensory perception (GO:0007600)	-	<0.01	0.000426	0.00866
Molecular Function	Glutamate receptor activity (GO:0008066)	+	22.96	7.33E-09	3.50E-07
	Nucleotide kinase activity (GO:0019201)	+	20.17	1.16E-06	3.70E-05
	Voltage-gated calcium channel activity (GO:0005245)	+	13.07	2.02E-03	3.51E-02
	Ligand-gated ion channel activity (GO:0015276)	+	9.8	3.71E-08	1.42E-06
	Voltage-gated ion channel activity (GO:0005244)	+	8.11	1.37E-04	2.91E-03
	Voltage-gated potassium channel activity (GO:0005249)	+	7.59	2.34E-03	3.44E-02
	Ion channel activity (GO:0005216)	+	7.13	1.86E-11	3.56E-09
	Cation channel activity (GO:0005261)	+	5.12	3.51E-03	4.19E-02
	Small GTPase regulator activity (GO:0005083)	+	4.64	2.25E-03	3.59E-02
	Transmembrane transporter activity (GO:0022857)	+	3.8	4.03E-09	3.84E-07
	Transporter activity (GO:0005215)	+	3.54	4.84E-09	3.08E-07
	GTPase activity (GO:0003924)	+	3.13	1.67E-03	3.19E-02
	Kinase activity (GO:0016301)	+	2.52	2.41E-03	3.29E-02
	Pyrophosphatase activity (GO:0016462)	+	2.38	3.84E-03	4.31E-02
	Catalytic activity (GO:0003824)	+	1.74	3.98E-06	1.08E-04
	Protein binding (GO:0005515)	+	1.64	0.00313	0.0399

* See Extended Data Table 1-1 for all Reactome pathway terms identified from the comparison between sham and acquisition groups.

**Table 2.** Results of PantherDB analysis for the comparison between sham and retrieval groups

PANTHER GO-Slim pathways		Over/Under	Fold enrichment	Raw *p* value	FDR (*q* value)
Cellular Component	Postsynaptic membrane (GO:0045211)	+	14.71	9.98E-09	7.99E-08
	Neuromuscular junction (GO:0031594)	+	11.03	3.83E-03	1.29E-02
	Proton-transporting ATP synthase complex (GO:0045259)	+	9.8	1.20E-03	4.51E-03
	Synapse (GO:0045202)	+	8.31	1.24E-12	7.96E-11
	Mitochondrial inner membrane (GO:0005743)	+	7.1	4.77E-09	5.08E-08
	Dendrite (GO:0030425)	+	7.08	1.49E-08	1.06E-07
	SNARE complex (GO:0031201)	+	6.06	5.75E-03	1.84E-02
	Neuron projection (GO:0043005)	+	5.33	2.61E-11	5.57E-10
	Cell junction (GO:0030054)	+	5.05	5.70E-05	2.60E-04
	Axon (GO:0030424)	+	4.68	1.31E-02	3.98E-02
	Cell projection (GO:0042995)	+	3.75	7.41E-09	6.77E-08
	Protein complex (GO:0043234)	+	2.31	8.91E-12	2.85E-10
	Cytoskeleton (GO:0005856)	+	2.2	5.35E-04	2.14E-03
	Membrane (GO:0016020)	+	1.99	1.14E-09	1.46E-08
	Integral to membrane (GO:0016021)	+	1.87	5.15E-04	2.20E-03
	Macromolecular complex (GO:0032991)	+	1.79	2.02E-07	1.17E-06
	Cytoplasm (GO:0005737)	+	1.76	5.38E-10	8.61E-09
	Plasma membrane (GO:0005886)	+	1.59	1.62E-03	5.77E-03
	Cell part (GO:0044464)	+	1.44	1.96E-07	1.25E-06
	Intracellular (GO:0005622)	+	1.37	2.18E-05	1.07E-04
Biological Process^*^	Growth (GO:0040007)	+	19.3	1.01E-03	8.82E-03
	Asymmetric protein localization (GO:0008105)	+	15.44	1.70E-03	1.34E-02
	Oxidative phosphorylation (GO:0006119)	+	10.89	2.67E-08	9.31E-07
	Pyrimidine nucleobase metabolic process (GO:0006206)	+	8.3	5.69E-04	6.31E-03
	JNK cascade (GO:0007254)	+	8.04	6.47E-04	6.32E-03
	Respiratory electron transport chain (GO:0022904)	+	7.35	3.03E-09	1.48E-07
	Generation of precursor metabolites and energy (GO:0006091)	+	6.03	1.52E-11	3.72E-09
	Neuron-neuron synaptic transmission (GO:0007270)	+	5.72	2.13E-05	3.25E-04
	Purine nucleobase metabolic process (GO:0006144)	+	5.63	4.04E-04	4.93E-03
	Glycolysis (GO:0006096)	+	5.51	1.15E-03	9.39E-03
	Mitochondrial transport (GO:0006839)	+	5.48	3.06E-03	1.86E-02
	Neurotransmitter secretion (GO:0007269)	+	4.98	1.46E-04	1.98E-03
	Cation transport (GO:0006812)	+	4.24	1.89E-03	1.44E-02
	Calcium-mediated signaling (GO:0019722)	+	4.14	2.14E-03	1.45E-02
	Mitochondrion organization (GO:0007005)	+	4	2.56E-03	1.65E-02
	Synaptic transmission (GO:0007268)	+	3.99	5.54E-10	4.51E-08
	Anatomical structure morphogenesis (GO:0009653)	+	3.68	6.22E-04	6.60E-03
	Protein targeting (GO:0006605)	+	3.47	5.29E-04	6.15E-03
	Ion transport (GO:0006811)	+	2.87	1.82E-05	2.96E-04
	Cell-cell signaling (GO:0007267)	+	2.86	3.92E-07	9.58E-06
Molecular Function^*^	Glutamate receptor activity (GO:0008066)	+	11.3	3.82E-07	9.12E-06
	Nucleotide kinase activity (GO:0019201)	+	10.29	1.32E-05	1.94E-04
	SNAP receptor activity (GO:0005484)	+	7.35	3.07E-03	2.66E-02
	Hydrogen ion transmembrane transporter activity (GO:0015078)	+	7.05	4.00E-06	8.48E-05
	Carbohydrate kinase activity (GO:0019200)	+	6.64	4.27E-03	3.26E-02
	Anion channel activity (GO:0005253)	+	5.25	3.61E-03	2.87E-02
	Ligand-gated ion channel activity (GO:0015276)	+	5.07	4.31E-06	8.24E-05
	Voltage-gated ion channel activity (GO:0005244)	+	4.14	2.14E-03	2.27E-02
	Ion channel activity (GO:0005216)	+	4.06	5.90E-09	3.76E-07
	Cation channel activity (GO:0005261)	+	3.58	2.49E-03	2.51E-02
	Microtubule binding (GO:0008017)	+	3.4	5.95E-03	4.37E-02
	Small GTPase regulator activity (GO:0005083)	+	3.39	1.12E-03	1.34E-02
	Oxidoreductase activity (GO:0016491)	+	3	5.89E-08	1.88E-06
	Kinase activity (GO:0016301)	+	2.79	2.71E-07	7.39E-06
	Calcium ion binding (GO:0005509)	+	2.79	2.80E-03	2.54E-02

* See Extended Data Table 2-1 for all GO-Slim pathway terms.

Extended Data Table 1-1 Results of Reactome pathway analysis for the comparison between sham and acquisition groups Download Table 1-1, XLSX file.

Extended Data Table 2-1 Results of GO slim analysis for the comparison between acquisition and retrieval groups Download Table 2-1, XLSX file.

The functional annotation clustering analysis provided by the DAVID bioinformatic database resulted in 54 annotation clusters for the comparison between sham and acquisition groups. Among them, 11 annotation clusters have an enrichment score >4.0 (Table 3; Extended Data Table 3-1). The functional clustering annotation terms “postsynaptic membrane” and “synapse” resulted in the highest enrichment scores (13.96 and 12.4, respectively). Moreover, the results also contain the terms regulation of excitatory postsynaptic potential, glutamate receptor, NMDA receptor, and neuronal membrane-associated guanylate-kinase-associated proteins. However, the functional annotation clustering analysis for the comparison between sham and retrieval groups did not result in any significant FDR (the lowest *q* = 1.72) with the highest enrichment score of 1.97; thus, no further analysis was considered.

**Table 3.** Results of DAVID Bioinformatics analysis for the comparison between sham and acquisition groups

	Functional annotation term summary^*^	Enrichment score	FDR (*q* value)			
Cluster			Median	SD	Lowest	Highest
1	Postsynaptic membrane	13.96	5.88E-13	0.055	1.65E-20	0.18
2	Synapse	12.40	6.63E-12	0.041	1.65E-20	0.092
3	Ion transport	9.54	3.25E-07	4.75E-07	1.49E-07	1.05E-06
4	Mitochondrial inner membrane	7.44	0.0154	0.093	2.25E-12	0.19
5	Positive regulation of excitatory postsynaptic potential	5.23	0.0014	0.848	3.79E-04	1.47
6	Neuronal membrane-associated guanylate kinases	5.16	0.0163	0.224	4.25E-04	0.46
7	PDZ and SH3 domains	4.91	0.0121	19.78	1.44E-07	65.75
8	Ionotropic glutamate and NMDA receptors	4.86	0.0272	0.859	3.01E-04	1.50
9	AMPA glutamate receptor complex	4.42	0.0104	5.28	0.0018	9.15
10	Glutamatergic synapses	4.34	0.1636	25.26	1.44E-07	100.0
11	Guanylate-kinase-associated protein	4.25	1.0843	0.682	4.25E-04	1.26

* See Extended Data Table 3-1 for detailed functional clustering annotation terms.

Extended Data Table 3-1 Results of functional clustering annotation analysis for the comparison between sham and acquisition groups. Download Table 3-1, XLSX file.

### IPA

IPA was conducted to identify the diseases and biological functions that are associated with behavior. From the comparison between retrieval and sham groups, functions of locomotion (*p* = 0.00028) and working (*p* = 0.00265) were detected (Extended Data Fig. 3-1). More behavior-associated functional annotations were detected from the comparison between the acquisition and sham groups (Fig. 3*A*; Extended Data Fig. 3-2). Interestingly, functions of memory, cognition and learning were significantly enhanced in the acquisition group while grooming, anxiety and emotional behavior were slightly, but significantly reduced. Statistically significant diseases and functions detected from the comparison between retrieval and sham groups include angiogenesis, apoptosis, necrosis, cognitive impairment and development of neurons (activation *z*-scores: –2.404, –2.205, –2.159, –1.941, and 1.514, respectively). tDCS before memory acquisition significantly enhanced plasticity of synapse, neurotransmission, synaptic transmission, synaptic transmission of nervous tissue, synaptic transmission of hippocampal cells, quantity of synaptic vesicles, function of neurons, branching of neurites, neuritogenesis, and long-term potentiation (LTP; activation *z*-scores: 2.36, 2.718, 2.701, 2.67, 2.762, 2.0, 2.0, 1.538, 1.291, and 1.259, respectively). Additionally, the acquisition group resulted in significant decreases in neurologic diseases, including hyperactive disorder, seizures, movement disorder, neurodegeneration, and cognitive impairment (activation *z*-scores: –3.679, –2.788, –2.015, –1.125, and –1.0, respectively).

10.1523/ENEURO.0311-19.2019.f3-1 Extended Data Figure 3-1 IPA diseases or functions annotation for the comparison between retrieval and sham groups. Download Figure 3-1, XLSX file.

10.1523/ENEURO.0311-19.2019.f3-2 Extended Data Figure 3-2 IPA diseases or functions annotation for the comparison between acquisition and sham groups Download Figure 3-2, XLSX file.

10.1523/ENEURO.0311-19.2019.f3-3 Extended Data Figure 3-3 Lists of IPA-generated networks for the comparisons between retrieval and sham groups and between acquisition and sham groups Download Figure 3-3, XLSX file.

**Figure 4.**
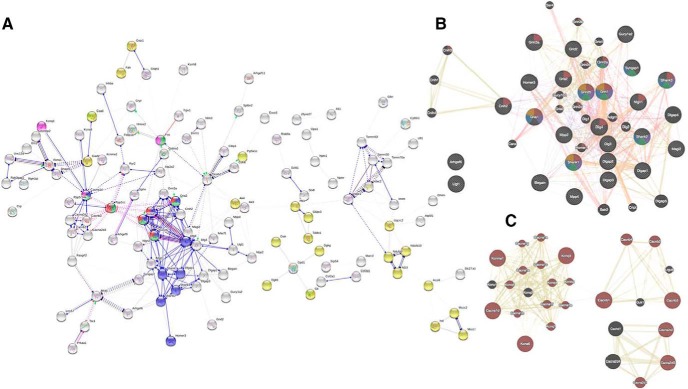
PPI network analysis. ***A***, PPI network for the significant molecules from the acquisition-and-sham comparison. Nodes were colored for functions of glutamatergic synapse (blue), metabolic pathways (yellow), dopaminergic synapse (green), LTP (red), and cholinergic synapse (pink). Disconnected nodes were excluded. ***B***, The first cluster network of MCL algorithm. ***C***, The second cluster network of MCL algorithm.

10.1523/ENEURO.0311-19.2019.f3-4 Extended Data Figure 3-4 Top regulators from the IPA upstream analysis for the comparisons between retrieval and sham groups and between acquisition and sham groups. Download Figure 3-4, XLSX file.

IPA generated multiple networks (Extended Data Fig. 3-3). Among them, only one network (Fig. 3*B*) identified from the analysis between retrieval and sham groups was associated with neurologic and psychological disorders. The analysis between the acquisition and sham groups resulted in 6 networks that are associated with the nervous system. When the six networks were merged into a larger network (Fig. 3*C*), molecules associated with memory (*p* = 1.65E-7), learning (*p* = 9.69E-16), and spatial learning (*p* = 9.09E-7) were most prevalent. The effects of top regulators were analyzed (Extended Data Fig. 3-4), but nervous system-associated regulators were not detected from the analysis between retrieval and sham groups. The analysis between the acquisition and sham group identified multiple top regulators that are associated with the nervous system. The network of regulators (APOE, BDNF, GSK3B, and SHANK3) targets 17 molecules in the dataset and are associated with function of neurons, memory, plasticity of synapse and synaptic transmission (consistency score: 8.731). Additionally, two networks including the nervous system regulator BDNF were identified and associated with synaptic transmission (total target molecule #: 5, consistency scores: –4.919 both). Top upstream regulators were also detected from the ingenuity upstream regulator analysis in IPA. The analysis between retrieval and sham groups identified MAPT, PSEN1, and APP (*p* values of overlap: 6.80E-16, 7.61E-10, and 3.77E-09, respectively) as top upstream regulators. PSEN1, MAPT1, APP, and L-dopa (*p* values of overlap: 2.95E-13, 3.03E-12, 1.04E-08, and 1.47E-08, respectively) were detected as the top upstream regulators for the comparison between the acquisition and sham groups.

### Network analysis

PPI networks were developed and analyzed to identify the effects of stimulation on protein signaling cascades. The PPI network for the statistically significant molecules detected from the retrieval compared to the sham group was not significant (average node degree: 0.53; average local clustering coefficient: 0.317; PPI enrichment *p* = 0.648). However, the PPI network from the acquisition group compared to the sham group had significant interactions (average node degree: 3.06; average local clustering coefficient: 0.404; PPI enrichment *p* < 1.0E-16; Fig. 4*A*). The PPI network was further clustered by the MCL method. The MCL cluster-1 network (Fig. 4*B*) was significantly related to the glutamate receptor signaling pathway (brown colored nodes; *q* = 2.68E-29), memory (purple colored nodes; *q* = 5.94E-07), learning (green colored nodes; *q* = 1.097E-06), cognition (blue colored nodes; *q* = 4.98E-06), and long-term memory (yellow colored nodes; *q* = 0.0001). The MCL cluster-2 network (Fig. 4*C*) was significantly associated with the function of voltage-gated channel activity (brown colored nodes; *q* = 4.03E-40). Network centralities were calculated for the MCL cluster-1 network to identify candidate molecules that may lethally affect to the network. Based on the subgraph centrality measure, the top five ranked proteins are DLG4, GRIN2a, SHANK1, GRIA1, and DLG3, and the expression of these proteins resulted in significant group difference between the acquisition and sham groups (*p* < 0.01).

**Figure 5.**
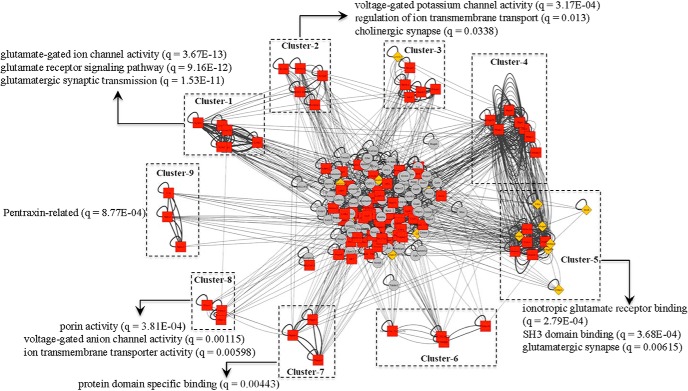
PSS network analysis. The BLASTP suite (BLASTP 2.8.0+) was used to search proteins and determine their sequence similarity. PSS network was created by using Cytoscape. The PSS network was clustered by using ClusterONE, and nine clusters were significantly identified. The edges were weighted by their sequence similarity and the enrichment analysis was performed for each of the nine clusters to identify specific signaling pathways of the clusters.

The BLASTP suite (BLASTP 2.8.0+) was used to search for proteins and determine their sequence similarity. PSS network was created by using Cytoscape, and proteins were clustered based on their sequence similarity. Among the 25 clusters identified, only nine clusters were statistically significant (*p* values for all clusters < 0.05; Fig. 5). However, clusters 3, 4, and 6 did not reach statistical significance for the enrichment analysis (PPI enrichment *p* > 0.05). As shown in Figure 5, each cluster is significantly related to a specific functional enrichment annotation. The clusters 1 and 5 are significantly related to glutamatergic pathways while clusters 2 and 8 are associated with voltage-gated channel activities. Functional annotations of protein domain specific binding and Pentraxin were significantly associated with the clusters 7 and 9, respectively. Based on the results of subgraph centrality measure for the PSS network, the top 10 ranked proteins are MACF1, SHANK1, ARHGEF6, DLG3, MPP2, MPP4, ARHGEF9, SHANK3, SHANK2, SHANK2 isoform 4 and MAGI2. The expression of the nine proteins (SHANK2 isoform 4 excluded) was significantly different between the acquisition and sham groups (*p* < 0.05).

## Discussion

In this study, we examined whether anodal tDCS could improve performance in a learning and memory task and the underlying protein modifications in the hippocampus, a brain region important for memory acquisition and recall. We have demonstrated that the timing of anodal tDCS is critical for memory performance. Anodal tDCS only improved memory performance when brain stimulation was applied before the acquisition period of the PAT. We further investigated the regulatory mechanisms for the memory enhancing effects of tDCS. Our results indicate tDCS induces changes in hippocampal synaptoneurosomes enhancing pathways related to cognition, learning, and memory. Additional analysis identified that the enhanced pathways are associated with specific receptor signaling and ion channel activity.

Our finding that anodal tDCS enhances cognitive performance when administered before memory acquisition is consistent with previous studies. A human-based study reported that tDCS enhances verbal memorization when anodal stimulation is applied during the memory encoding period (Javadi and Walsh, 2012). Another recent study shows that anodal tDCS delivered during the memory encoding period, but not the retrieval period, enhances memory (Medvedeva et al., 2019). Fertonani et al. (2014) also found that the timing of stimulation in relation to a cognitive task significantly affected performance. Together, previous studies and our current work suggest that tDCS-induced memory enhancement is dependent on the timing of tDCS administration.

To understand the underlying mechanisms for anodal tDCS-induced memory enhancement, we profiled proteins in the hippocampal synaptoneurosomes and analyzed the proteomic data. Compared to the sham treatment group, there were significant differences in protein abundances for the acquisition group (184 proteins). The PCA and hierarchical clustering analysis show a clear separation in distribution for the sham and acquisition groups. Enrichment and pathway analyses for the statistically significant proteins between the sham and acquisition group show changes in hippocampal protein regulation associated with synaptic neurotransmission and transporters. Using multiple database analyses from PantherDB and David bioinformatics (Tables 1 and 3), we further identified pathways involving glutamate receptor and ion channel signaling. Glutamate receptors and ion channels regulate neuronal activity and synaptic plasticity, which underlie neurologic processes of learning and memory (Gasque et al., 2006; Voglis and Tavernarakis, 2006; Gecz, 2010; Queenan et al., 2017; Foster et al., 2018). Although the underlying mechanisms of tDCS on learning and memory remain under investigation, our data show that anodal tDCS applied before the memory acquisition period modifies the regulation of hippocampal glutamate signaling and ion channel activity ultimately resulting in enhanced memory performance in rats.

We used IPA to understand hippocampal biological functions associated with learning and memory. Similar to the enrichment and pathway analysis results, the comparison between the acquisition and sham groups identified significant enhancements of memory, cognition, and learning while negative behaviors, such as anxiety, were significantly reduced. Our data show that anodal tDCS applied before the acquisition period modifies synaptic proteomic regulation in a manner associated with multiple electrophysiological and molecular pathways such as neuritogenesis, LTP, branching of neurites and synaptic transmission, which consequently enhance learning and memory. There is evidence that these pathways possibly explain the beneficial effects of tDCS on cognition. For example, tDCS has been reported to modulate LTP, which is well known to enhance cognition (Ranieri et al., 2012). Additionally, the networks developed by IPA contain many molecules that have been known to be associated with learning and memory. APOE, BDNF, GSK3B, and SHANK3 were identified as candidate regulators of the network to control memory and synaptic plasticity. PSEN1, MAPT1, APP, and L-dopa were identified as upstream regulators for significant proteins detected from the comparison between sham and acquisition groups. Most of these candidate regulators have been reported to be associated with cognition: APOE (Risacher et al., 2015; Sinclair et al., 2015), BDNF (Poo, 2001), PSEN1 (Kennard and Harrison, 2014), APP (Tamayev and D'Adamio, 2012), and L-DOPA (Murer and Moratalla, 2011). A recent study shows that tDCS induces BDNF expression and consequently enhances memory (Cocco et al., 2018). Based on the results of the Ingenuity upstream regulator analysis in IPA, our study is the first to suggest the following potential candidate regulators for anodal tDCS effects on learning and memory: GSK3b, SHANK3, and MAPT1. Most research on the role SHANK3 in the brain is related to autism (Yi et al., 2016). Recently, Duffney et al. (2013) showed that SHANK3 is associated with NMDA receptor function, suggesting a potential role for SHANK3 in learning and memory. Currently, there are no published reports linking GSK3b with tDCS-induced learning and memory, but a recent study reported that GSK3b is associated with Alzheimer’s disease (Jayapalan and Natarajan, 2013). We cannot find any research that investigates roles of MAPT1 in learning and memory, but there is evidence that a MAPT mutation may be indirectly associated with cognitive dysfunction observed in dementia (Iyer et al., 2013). More work is needed to elucidate the roles of these novel candidate molecules in learning and memory.

To identify significant protein networks for tDCS-induced effects on proteomic modifications of the hippocampal synapse, we conducted network analysis. The PPI network for the acquisition group compared to sham group is significantly associated with learning, memory, cognition, long-term memory, and glutamate receptor signaling pathways. Significant glutamate signaling pathways and ion channel activity were also identified from the PSS network. A relationship between ion channel activity and memory performance has been previously reported (Cavallaro, 2004) and it showed higher ion channel activity in animals is significantly associated with better memory performance as measured by the PAT. Glutamate receptor signaling pathways are also known to be dynamically regulated during learning and memory (Tronson and Taylor, 2007; Poo et al., 2016; Foster et al., 2018). Our studies have identified significant glutamate signaling and channel activity for the acquisition and sham group comparison using multiple analyses including enrichment and pathway analyses, IPA and PPI and PSS networks providing additional evidence for the mechanism of tDCS effects on hippocampal synaptoneurosomes. Therefore, our data suggest that anodal tDCS applied before the acquisition period modifies glutamate signaling and ion channel activity and consequently enhances memory performance. Our results identifying glutamatergic signaling as a mechanism for tDCS-induced effects on learning are supported by previous reports (Rossini et al., 2015; Giordano et al., 2017; Stafford et al., 2018). Our work is the first to use proteomics to suggest possible molecular effects associated with tDCS-enhancing memory acquisition by modifying glutamate signaling and ion channel activity pathways in rat hippocampal synaptoneurosomes.

From the PPI and PSS networks, we identified key molecules that lethally affect the protein networks. Key molecules in the networks identified by subgraph centrality measure include SHANK, DLG, GRIN, and GRIA proteins. Although future studies are needed to investigate the effects of these key players in the network, our work supports possible roles of these proteins in learning and memory. The function of glutamate receptor (Grin2a and Gria1) in learning and memory is well understood. Additionally, recent studies suggest SHANK may mediate the activity of glutamate receptors in neurons (Bertaso et al., 2010; Ha et al., 2018) and may play a role in synapse formation and plasticity (Kilinc, 2018). SHANK was also identified as one of the regulators in the network from the IPA suggesting that anodal tDCS modulates SHANK and subsequently affects synaptic plasticity and transmission in hippocampal neurons. Additionally, SHANK and DLG proteins were also identified as critical molecules in the PSS network.

Bioinformatics and network analyses were completed for the comparisons of the retrieval group to the sham and acquisition group even through the behavioral data were not significant. Compared to the sham treatment group, there were significant differences in protein abundance for the retrieval group (82 proteins) Furthermore, the candidate regulators suggested from the Ingenuity upstream regulator analysis in IPA for the comparison between the sham and acquisition group (see above) did not result in a statistically significant difference. Additionally, the clustering analyses did not show a clear separation between the retrieval group and the sham and acquisition groups. Comparison of the acquisition group to the retrieval group using enrichment and pathway analyses, generated significant effects of hippocampal protein regulation of glutamate receptor pathways and ion channel pathways. Unique pathways identified for the acquisition and retrieval group comparison include pyrophosphatase activity and SNAP receptor activity. Many similar pathways were identified from the acquisition and sham versus the acquisition and retrieval group comparisons (Tables 1, 2), suggesting similarity between the sham and retrieval groups. More time poststimulation may be needed for the molecular modifications in the retrieval group to show a clear separation from the sham group.

In summary, we investigated whether anodal tDCS could affect memory acquisition, recall, or both, and the underlying molecular modifications in hippocampal synaptoneurosomes. We found that anodal tDCS administered before acquisition significantly enhanced memory performance potentially by enhancing the abundance of proteins associated with glutamate signaling and ion channel activity in hippocampal synapses. Our results also suggest potential molecular targets for the effects of anodal tDCS on memory performance. Our work contributes to the understanding of the regulatory molecular mechanisms of tDCS-induced memory enhancements. However, because the focus of our study is to observe molecular differences in hippocampal synapses and correlate proteomic changes with rodent cognitive performance, we should point out that our data do not report any cause-and-effect relationship between proteomic modifications, tDCS, and cognitive performance. Thus, future studies are needed to investigate the causal effects of the proteins identified from our study. Increased understanding of the mechanisms for the effects of tDCS on memory enhancement will facilitate future applications of brain stimulation.

## Synthesis

Reviewing Editor: Alfonso Araque, University of Minnesota Decisions are customarily a result of the Reviewing Editor and the peer reviewers coming together and discussing their recommendations until a consensus is reached. When revisions are invited, a fact-based synthesis statement explaining their decision and outlining what is needed to prepare a revision will be listed below. The following reviewer(s) agreed to reveal their identity: Greg Kronberg, Rocio Gomez-Pastor. Note: If this manuscript was transferred from JNeurosci and a decision was made to accept the manuscript without peer review, a brief statement to this effect will instead be what is listed below.

The reviewers have found merit and interest on the findings reported. I concur with the reviewers' comments expressing the interest of the study. However, they also expressed some concerns about several issues that need to be addressed and clarified. I also consider pertinent the concerns expressed.

I believe that addressing those concerns, which will definitely improve the manuscript, is necessarily required to firmly validate the results and support the conclusions.

Specific comments of the reviewers:

Reviewer 1.

Here the authors study the effect of tDCS either during the acquisition or retrieval of passive avoidance memory in rats. They demonstrate an interesting behavioral result, namely that tDCS can enhance learning only when applied during the acquisition phase. An ongoing question in the field is when to apply stimulation relative to a behavioral task, and this result may contribute to answering this question. The authors then look proteomic analyses of isolated synaptoneurosomes from animals after learning. I am unfamiliar with many of the analytical tools that were used to analyze this data, and so I cannot comment on their validity. However, under the assumption that their methods are sound, the manuscript is well written and this study makes a valuable contribution to field . First by helping to answer the timing question described above, and second by providing proteomic data that may help identify pathways that are affected by tDCS.

I have only minor concerns about the interpretation of the proteomic results. It seems that the change in protein levels due to tDCS could be explained by altered hippocampal LTP, which is already a well-studied effect of this tDCS protocol. The authors should discuss the extent to which this is a likely explanation. Moreover, it should be made explicit that these methods cannot distinguish between proteins that mediate the effects of tDCS on learning and those that are upregulated as a result of the enhanced learning itself. While I think the proteomic data are valuable, as they can serve to constrain mechanistic models, the mechanistic insight gained here may unfortunately very limited beyond what is already known about the effects tDCS on synaptic plasticity.

Reviewer 2.

The work performed in this study provides a nice set of data exploring the proteomic effects of anodal tDCS, showing the importance of applying this procedure prior to memory acquisition and the alterations in specific set of proteins with relevance in memory and cognition. I find this study very interesting in advancing our knowledge on how anodal tDCS exerts its effects in memory and cognition.

Some minor issues have been identified that need to be considered prior to publication.

-Lane 416. The authors state that their study is the first to identify GSK3b, SHANK3 and MAPT1 as candidate regulators for anodal tDCS and its effect on memory. Based on the data provided in the mansucript it is unclear how they authors came up with these candidates. Just because these 3 proteins have somewhat been previously related to cognition and or memory and also tDCS, the provided data is not enough to support those proteins as candidates for regulating anodal tDCS. Please explain/clarify what is the criteria used to select these 3 proteins as candidate regulators.

-The authors indicated that comparison of the sham and retrieval group reflected changes in 82 proteins but clustering analyses did not show a clear separation in functional groups. Do any of those 82 proteins correspond to their suggested candidate regulators despite not observing significant functional groups in their analysis? Please include this information in the manuscript.

-In lanes 442-443 the authors state that this study provides the first molecular evidence for tDCS enhancing memory acquisition by modifying glutamate signaling and ion channel activity. However, the evidence provided only shows correlation with enhanced expression of proteins in these pathways and it is not enough to suggest that this is the first evidence for a causal role. I understand that the purpose of the current manuscript is not studying the causal effect of these pathways. Therefore, without providing new data I suggest soften that sentence to better reflect the correlative nature of the study and not causality.

-Number of animals used for the study is completely necessary. Please include those in the Methods sections and figure legends.

-In lane 449 the authors indicate...“ SHANK may mediate glutamate receptors in neurons...”. This sentence is unclear. Does SHANK mediate expression, activity, membrane localization...?

-In lane 452 the authors indicated that tDCS modulates Shank (in small letters). Are they suggesting modulation of protein levels or gene expression. Since they authors refer to this protein in capital letters SHANK I would suggest to keep consistency in the nomenclature or explain whether they refer to gene and/or protein.

-In the description of all Tables titles the authors indicate “...significant proteins from....”. When reading these title it is expected to find a list or protein names rather than functional annotation terms or GO. In Tables 1,2 and 3 no proteins are listed but instead GO terms. Please modify the title of the tables accordingly to better reflect the information provided.

-In the methods section the authors described that animals are immediately euthanized after behavioral tests for brain tissue collection. Please describe the method use to euthanize the animals.

-Figure 2. Panel A Legend should include information about the significance of asterisks (*). Are they p-values? Statistical information should be included in panels B and C. For panel E, protein names are really small and very difficult to read. I suggest to modify the figure to better read those names.
