# Supplementary figures and images for: Noninvasive Brain Stimulation Enhances Memory Acquisition and Is Associated with Synaptoneurosome Modification in the Rat Hippocampus
Source: eNeuro. 2019 Dec 3;6(6):ENEURO.0311-19.2019. doi: 10.1523/ENEURO.0311-19.2019 (PMC6900464; doi:10.1523/ENEURO.0311-19.2019)

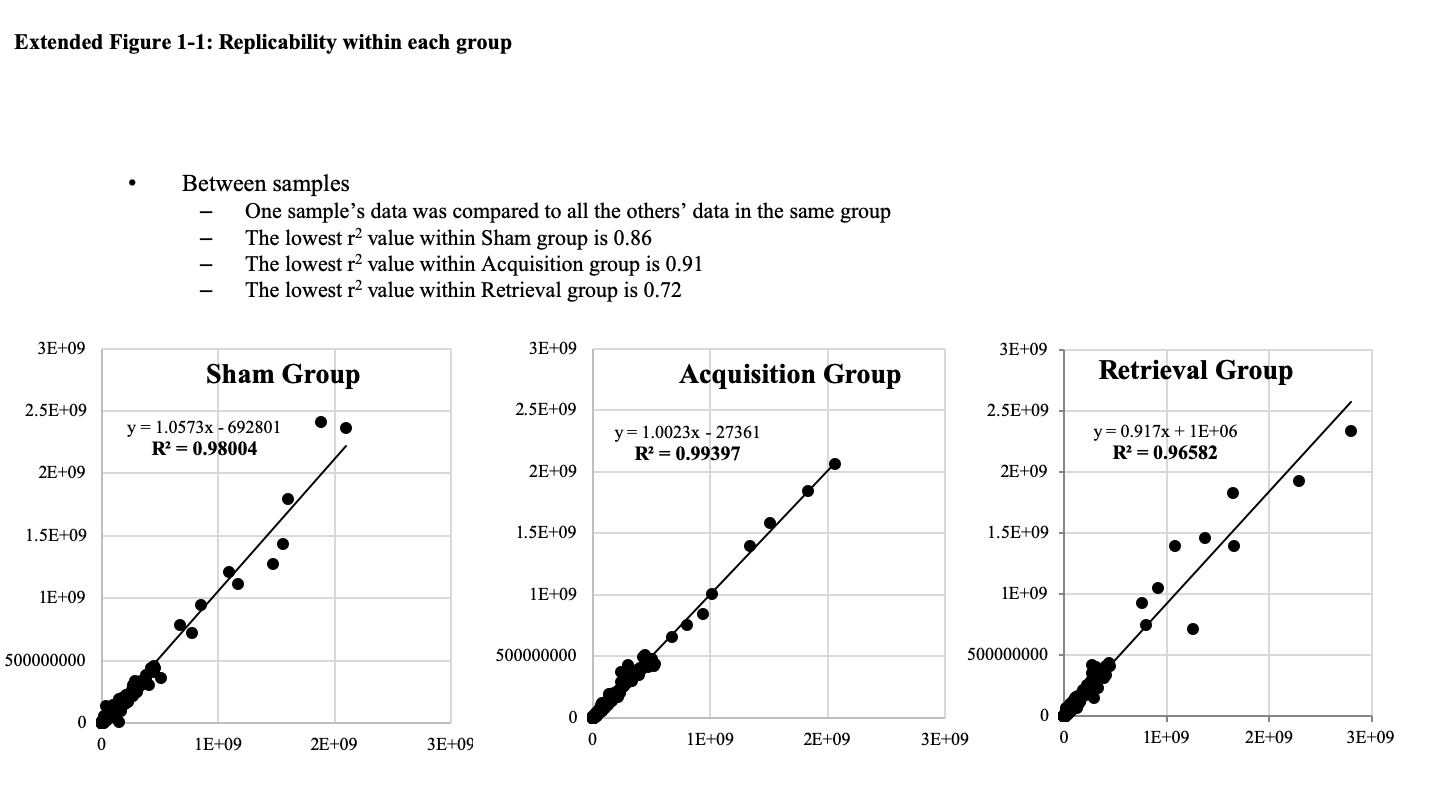

Supplement: Extended Data Figure 1-1 — Replicability of proteomic abundance data within each group. The abundance values within each group were analyzed and the lowest r2 for the sham, acquisition and retrieval groups were 0.89, 0.91, and 0.72, respectively. Download Figure 1-1, TIF file. [file sup_enu-eN-NWR-0311-19-s02.tif]

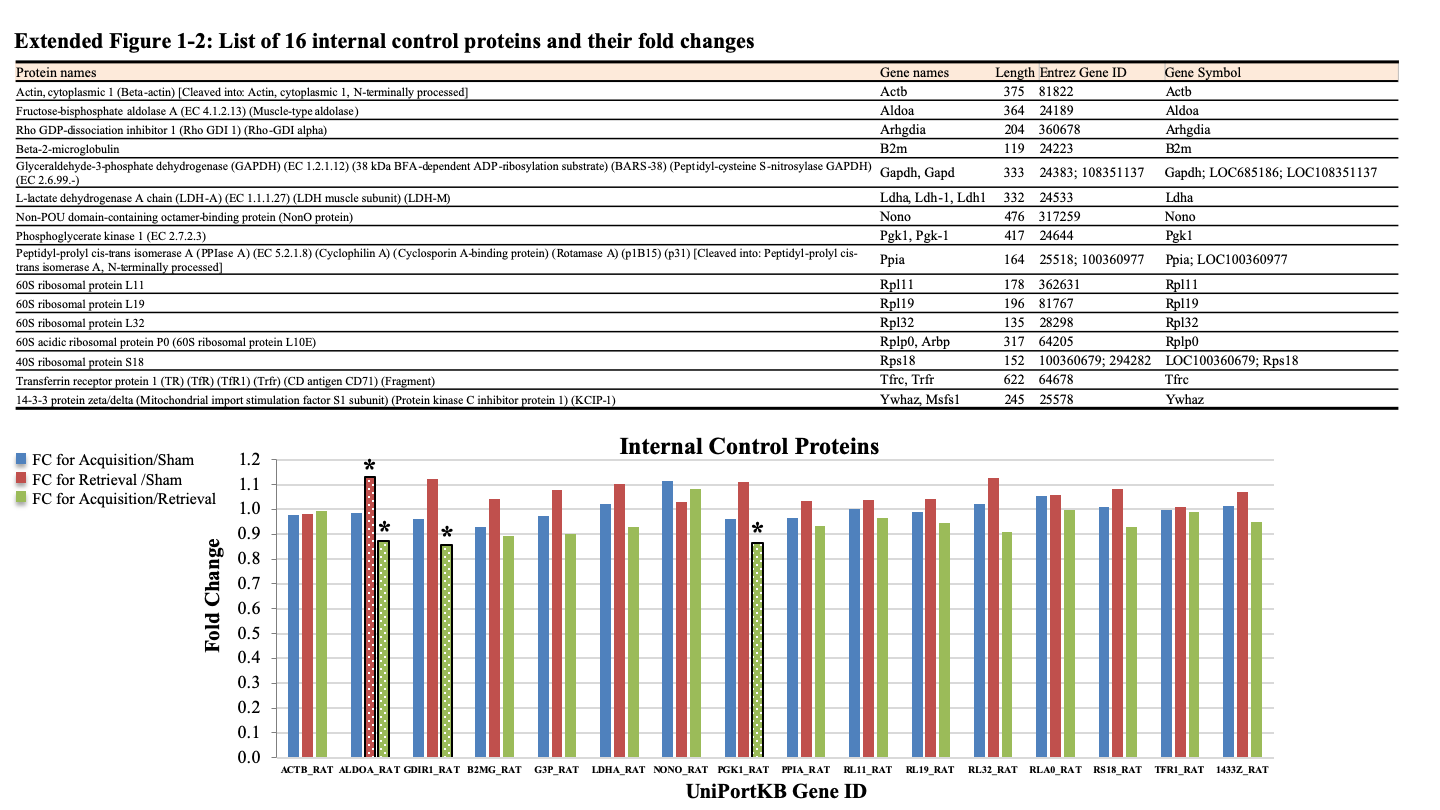

Supplement: Extended Data Figure 1-2 — Abundance comparison of 16 internal control proteins between groups. None of all 16 internal control proteins resulted in no significant difference between acquisition and sham groups. The comparison between retrieval and sham groups, one internal control protein (Aldoa) showed a significant group difference. The abundance data of three internal control proteins (Aldoa, Gdir1, and Pgk1) resulted in a significant group difference between acquisition and retrieval groups. Download Figure 1-2, TIF file. [file sup_enu-eN-NWR-0311-19-s03.tif]

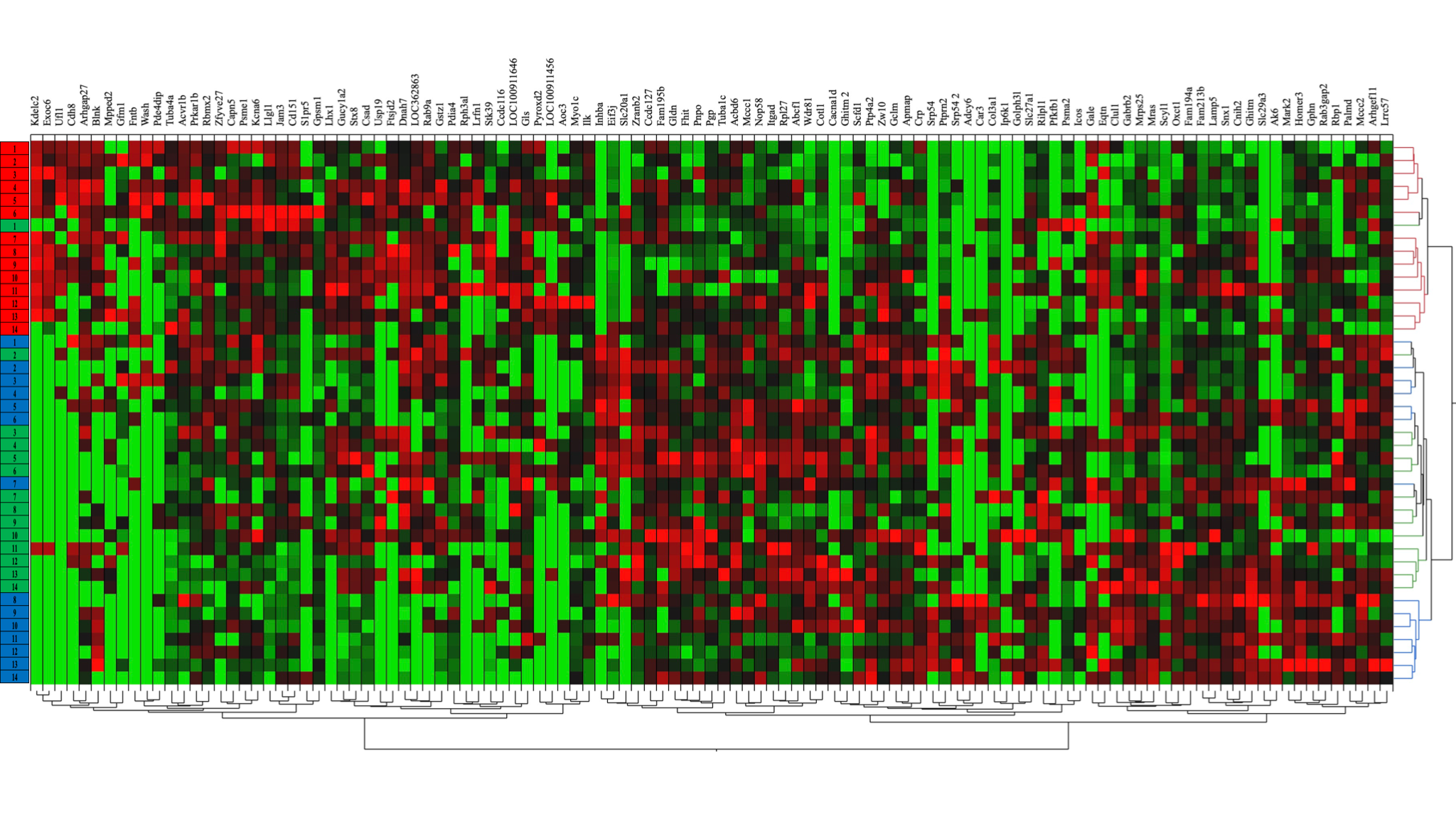

Supplement: Extended Data Figure 2-1 — Hierarchical clustering analysis with proteomics data show the distribution of each sample across the groups. Download Figure 2-1, TIF file. [file sup_enu-eN-NWR-0311-19-s04.tif]
